# 

**Published:** 1880-10

**Authors:** 


### BOOKS AND PAMPHLETS RECEIVED.

### BOOKS.

Index Catalogue of Library of Surgeon General's Office, U. S. Army; Vol I. Washington; 1880.

Organic Materia Medica. By L. E. Sayre, PH.G. Detroit; 1880.

Treatise on Therapeutics; Vol. II. By A. Trosseau and H. Pidoux. Translated by B. F. Lineslee, M.D. Wood's Library; 1880.

The Skin—in Health and Disease. By L. D. Bulkley, M.D.

The Brain as an Organ of the Mind. By H. C. Bastian, M.D., F.R.S.

Functional Nervous Diseases. By L. Putzel, M.D. 1880.

Dose Book and Anatomist. By C. H. Leonard, M.D. 1880.

Geo. P. Rowell & Co.'s American Newspaper Directory. 1880.

Practitioner's Hand Book of Treatment. By J. Milner Fothergill, M.D. 1880

Contributions to Orthopœdic Surgery. By J. C. Hutchison, M.D. New York; 1880.

Tumors of the Mammary Gland. By S. W. Gross, M.D. Philadelphia; 1880.

The Microscopist. By J. H. Wythe. Philadelphia; 1880.

Diseases of Women. By Graily Hewitt, M.D. Philadelphia; 1880.

### PAMPHLETS.

Seventeenth Annual Report of New York Society for Relief of Ruptured and Crippled. May, 1880.

Case of Probable Abscess of Brain. By Frank Allport, M.D.

Iritis and Some of its Dangers. By S. J. Jones, M.D. (Reprint.)

Fifteenth Annual Report of Chicago Hospital for Women and Children. March 1, 1880.

Transactions State Medical Society of Arkansas, fifth annual session. 1880

Pregnancy Vomiting. By J. Marion Sims, M.D.

Report of Indiana State Health Commission. 1880.

Anæsthesia by Ethyl Bromide. By H. Augustus Wilson, M.D.

Lunacy Reform. By E. C. Seguin, M.D.

Peptonized Milk as Food for Infiants and Invalids. By R. J. Nunn, M.D.

Practical Hints Relating to Yellow Fever Prevention. By R. B. S. Hargis, M.D.

Minutes of Twenty-fourth and Twenty-fifth Annual Meetings of Kentucky State Medical Society. 1879 and 1880.

Reports and Resolutions Relating to Sanitary Legislation.

Lacerations of the Neck of the Uterus. By A. R. Jackson, M.D.

Eleventh Report of State Board of Health of Massachusetts. 1879.

Transactions Medical and Chirurgical Society of Maryland. 1880.

Proceedings of Connecticut Medical Society. 1880.

---

IN the *British Medical Journal* for Dec. 25, 1875, is recorded a case of diabetes insipidus successfully treated with ergot, after the failure of jaborandi and other remedies. Half a drachm of the fluid extract of ergot every three hours reduced the urine in twenty-four days from twenty pints to a pint and a half; increased its specific gravity from 1.002 to 1.017 ; and removed the excessive thirst and other distressing symptoms of over two years' standing.

A few days ago the reporter of the case accidentally met the patient, who told him he had never had a day's illness since he left the hospital four and a half years ago ; his urine was normal in quantity, and he did not suffer from thirst. He was strong and well in every way, and able to do a good day's work.

SOCIETY MEETINGS.

Chicago Medical Society—Mondays, Oct. 4 and 18.

West Chicago Medical Society—Mondays, Oct. 11 and 28.

Biological Society—Wednesday, Oct. 6.

CLINICS.

MONDAY.

Eye and Ear Infirmary—2 p. m., Ophthalmological, by Prof. Holmes; 3 p. m., Otological, by Prof. Jones.

Mercy Hospital—2 p. m., Surgical, by Prof. Andrews.

Rush Medical College—2 p. m., Dermatological and Venereal, by Prof. Hyde.

Woman's Medical College—2 p. m., Dermatological and Venereal, by Prof. Maynard; 3 p. m., Diseases of the Chest, Prof. Ingals.

TUESDAY.

Cook County Hospital—2 to 4 p. m., Medical and Surgical Clinics.

Mercy Hospital—2 p. m., Medical, by Prof. Quine.

WEDNESDAY.

Chicago Medical College—2 p. m., Eye and Ear, by Prof. Jones.

Rush Medical College—2 p. m., Medical, by Dr. Bridge; 3 p. m., Ophthalmological and Otological, by Prof. Holmes; 3:30 to 4:30 p. m., Diseases of the Chest, by Dr. E. Fletcher Ingals.

THURSDAY.

Chicago Medical College—2 p. m., Gynæcological, by Prof. Jenks.

Rush Medical College—2 p. m., Diseases of Children, by Dr. Knox; 3 p. m., Diseases of the Nervous System, by Prof. Lyman.

Eye and Ear Infirmary—2 p. m., Ophthalmological, by Dr. Hotz.

Woman's Medical College—3 p. m., Surgical, by Prof. Owens.

FRIDAY.

Cook County Hospital—2 to 4 p. m., Medical and Surgical Clinics.

Mercy Hospital—2 p. m., Medical, by Prof. Davis.

SATURDAY.

Rush Medical College—2 p. m., Surgical, by Prof. Gunn; 3 p. m., Orthopœdic, by Prof. Owens.

Chicago Medical College—2 p. m., Surgical, by Prof. Isham; 3 p. m., Neurological, by Prof. Jewell.

Woman's Medical College—11 a. m., Ophthalmological, by Prof. Montgomery; 2 p. m., Gynæcological, by Prof. Fitch.

Daily Clinics, from 2 to 4 p. m., at the Central Free Dispensary, and at the South Side Dispensary.

